# Feasibility of Using Mobile Technology to Improve Physical Activity Among People Living with Diabetes in Asia

**DOI:** 10.31372/20200504.1110

**Published:** 2021

**Authors:** Nada Lukkahatai, Pratum Soivong, Dongmei Li, Phakjira Jaiman, Supornphan Thamkaew, Duenapen Chaiwong, Nutchari Hiranlalit, Jillian Inouye

**Affiliations:** a School of Nursing, Johns Hopkins University, Baltimore, Maryland, United States; b Faculty of Nursing, Chiang Mai University, Chiang Mai, Thailand; c University of Rochester, Rochester, New York, United States; d Medical Unit, U.S. Embassy Bangkok, Thailand; e Nakornping Hospital, Chiang Mai, Thailand; f Hangdong Hospital, Chiang Mai, Thailand; g John A. Burns School of Medicine, University of Hawai‘i, Hawai‘i, United States

**Keywords:** physical activity, mobile health, wearable device, diabetes, Thailand

## Abstract

**Background:** Chronic conditions such as diabetes (DM) and cardiovascular disease are associated with disability and poor quality of life. Asians are 30% more likely than non-Hispanic Whites to develop type 2 DM. The important roles of wearable technological applications or devices in maintaining individuals’ motivation to attain their physical activity (PA) goal have been reported. However, evidence of the feasibility and impact of the mobile technology on the PA in Thailand is limited. This study aims to examine the feasibility and the impact of the immediate performance feedback feature of the wearable device on PA.

**Methods:** This pilot study recruited persons aged 18 or older with diabetes from two diabetes clinics in Chiang Mai, Thailand. Participants were randomly assigned into three groups: the aware group (AW: can see the step count on the device screen), the unaware group (UW: the device screen was completely covered), and the control group (usual care). Participants in the AW and UW groups were asked to wear the device for 2 full days while the usual care group did not wear the device. All participants completed a questionnaire package at their first study visit. Data analysis of univariate and multivariate general linear models were conducted using SAS version 9.4 (SAS Institute Inc., Cary, NC). All significance levels were set at 5%.

**Results:** One hundred and fourteen participants age ranged from 39 to 75 years old were included in this analysis. The majority were female (*n* = 86, 69%) and married (*n* = 103, 82%). No adverse events were reported among device users. Within 2 days, there were less than 10% missing data and more than 70% of participants liked the devices mainly because they could see the step count. More than 63% of participants who wore the device had an average of steps greater than 10,000 per day. Although the number of steps and sleep hours were not significantly different between the AW and UW groups, 68% of the AW participants found that the device help them with their exercise.

**Discussion:** The results demonstrate the feasibility of the use of the wearable device among people living with chronic conditions. Participants found that the step count screen provided immediate physical performance feedback that was helpful with their exercise. The behavioral changes, however, could not be examined due to the short duration of the usage. Future studies that require longer device usage in larger sample sizes are needed.

## Introduction

Diabetes mellitus (DM) is a major chronic health problem associated with disability and poor quality of life (Bourdel-Marchasson, Helmer, Fagot-Campagna, Dehail, & Joseph, 2007). According to the U.S. National Health Interview Survey, Asians are 30% more likely to develop diabetes than non-Hispanic whites (Lee, Brancati, & Yeh, 2011). DM is one of the top five leading causes of death among Asian and Pacific Islander populations in the United States (Heron, 2013). In Thailand, DM is one of the leading causes of death among Thai adults (Center for Disease Control and Prevention, 2013), and the second cause of death among Thai women (Deerochanawong & Ferrario, 2013).

Physical activity (PA) is known to effectively manage and prevent many chronic conditions including diabetes (Hoffmann et al., 2016) and to reduce hemoglobin A1c (HbA1C) (Khongruangrat, Vannarit, & Lukkahatai, 2012) and symptoms (pain, fatigue, and sleep difficulty) (Beland et al., 2020; Cox, Coombes, Keating, Burton, & Coombes, 2020; Dodd et al., 2010; Hoffman et al., 2013; Spector, Deal, Amos, Yang, & Battaglini, 2014). Despite the well-known health benefits of regular exercise, many adults living with diabetes do not participate in exercise and are less physically active than the general population (Dnes et al., 2020; Pinto & Ciccolo, 2011). Low engagement rates with exercise regimens and a lack of motivation are barriers in most exercise and PA programs (Advika, Idiculla, & Kumari, 2017; Clarke et al., 2015; Lascar et al., 2014; Malone, Barfield, & Brasher, 2012). The important roles of mobile technologies and devices in maintaining individuals’ motivation to attain their PA goals have been reported (Cushing, Jensen, & Steele, 2011; Jones et al., 2014; Verbeken, Braet, Goossens, & van der Oord, 2013). These wearable devices were recently used as a tool for real-time data collection, sharing immediate feedback, and reminder messaging in many PA programs (Nguyen et al., 2017; Sanders et al., 2016; Simunek et al., 2019). However, it is unclear whether features such as revealing the number of steps alone would serve as a motivator to the wearers to increase their daily PAs. Moreover, the feasibility and effect of the real-time step count feature of a wearable device on PA have not been tested among patients with diabetes in Thailand.

Based on the biopsychosocial model, health was influenced by social, psycho-behavioral, and biological factors. Studies have reported that culture, religion, socioeconomic status, and family support influence self-management and PA in persons with chronic conditions such as diabetes (Lundberg & Thrakul, 2012, 2013; Sowattanangoon, Kochabhakdi, & Petrie, 2008, 2009; Sriussadaporn, Ploybutr, Nitiyanant, Vannasaeng, & Vichayanrat, 1998). Studies have reported that Thailand’s unique social structure, culture, and religion such as the importance of rice in Thai cuisine, extended family lifestyle, and religious beliefs influence how Thai patients manage their diabetes (Kangwanrattanakul, Gross, Sunantiwat, & Thavorncharoensap, 2019; Sowattanangoon et al., 2009). However, the influential factors of PA among this population remain unclear. In Western culture, immediate performance feedback while using technology has an impact on individuals’ PA. It is unknown whether this feature would have a similar effect on health behaviors and PA in Thailand.

**Figure 1:**
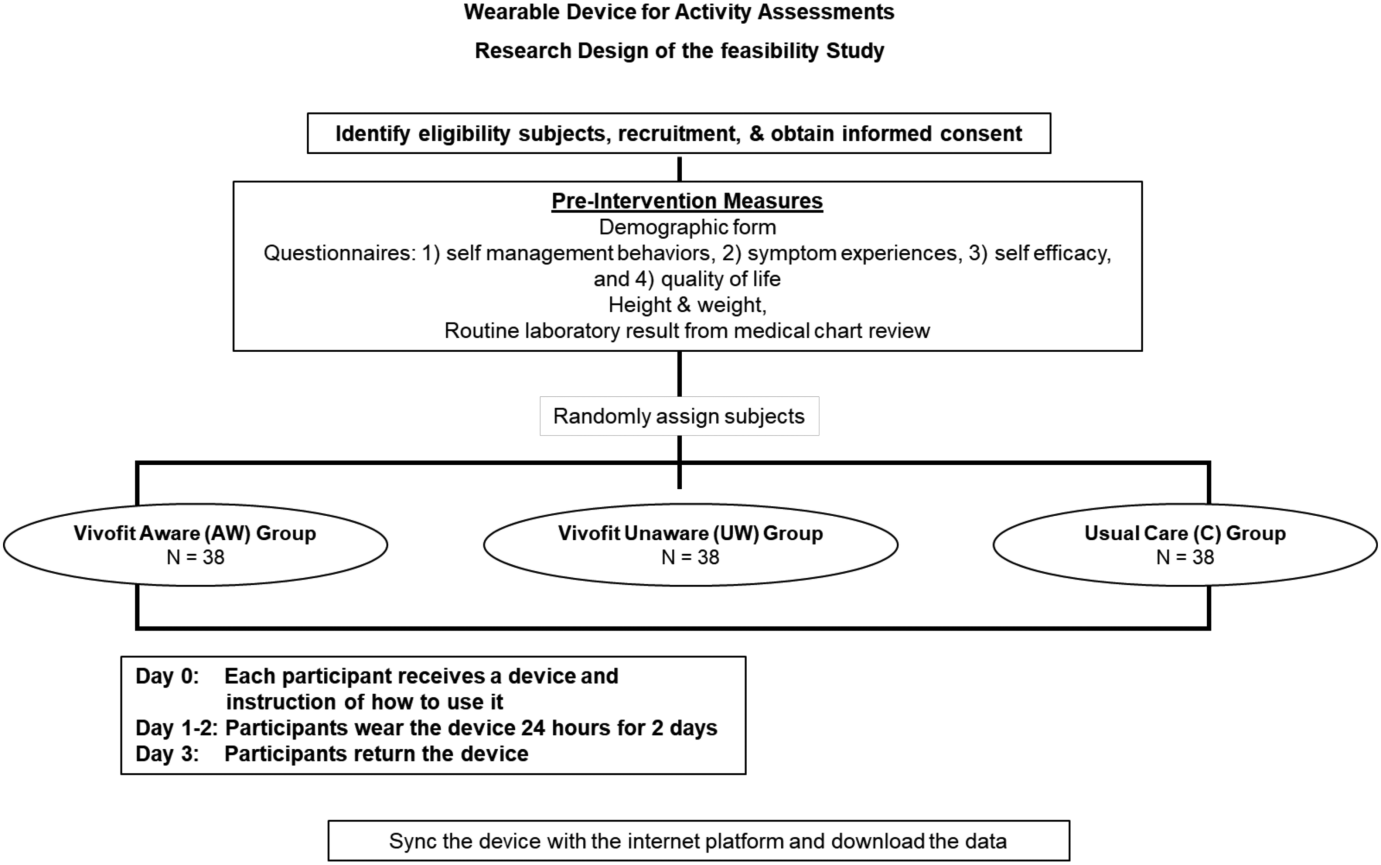
Data collection process.

This three-arms trial consisted of two intervention groups: Vivofit aware (AW) and Vivofit unaware (UW) groups and a control (C) group (Figure 1). While the control group continued to receive usual care, participants in both intervention groups were asked to wear a wearable device for 2 full days. Participants in the AW group were able to see the daily step count on the device screen. In the UW groups, the wearable device screens were painted in black ink so the participants could not see the step counts.

The study aimed to: (1) examine the feasibility of wearable technology among participants who used the device (AW and UW), (2) examine the impact of the immediate performance feedback feature (revealing the step counts screen) of the device on PA levels and sleep by comparing step counts and hours of sleep between the AW and UW groups, and (3) explore the differences in self-management behaviors, quality of life, symptoms (fatigue and pain), and self-efficacy among the three groups (AW, UW, and C). We hypothesized that: (1) the wearable technology is feasible and acceptable among Thai patients with diabetes; (2) by providing immediate physical performance feedback (daily step counts), individuals in the AW (Vivofit aware) group would have higher PA and longer sleep duration than those in the UW (Vivofit unaware) group; and (3) participants in the AW group would have better self-management behaviors, quality of life, symptoms, and self-efficacy than those in the UW and C groups. Information obtained from this study will be used to guide the long-term strategies and interventions that are planned to empower Thai patients to manage their chronic conditions and symptoms.

## Methods

### Study Design

The study is a pilot study using a wearable device (Garmin Vivofit) in a two- full days period. The researchers used the internet platform to extract step counts and sleep data from the wearable. Participants were asked to complete a questionnaire package to measure self-management, the experience of symptoms, self-efficacy, and quality of life.

### Participants

We recruited 114 Thai heritage adults ages 18 years or older, diagnosed with type 2 diabetes (T2DM), who were followed up at two local health-promoting rural area hospitals in the Northern Thailand region (Chiang Mai). Participants with serious health conditions and severe complications from diabetes (e.g., renal failure, severe earand nerve damage) were excluded from the study.

### Sample Size and Power Analysis

Power analysis was conducted based on the primary outcomes of bivariate correlations between PA and biopsychosocial measures (Pearson’s *r*) using the Proc Power procedure in SAS version 9.4 (SAS Institute, Cary, NC). Assuming a conservative medium effect size (pho = .3), we needed 84 subjects to achieve a power of 0.80 at the 5% level of significance. With a conservative attrition rate of 10%, we needed to recruit at least 94 subjects into our study to maintain 80% power. Allowed by our resources, we planned to recruit 114 subjects into our study and a sample size of 114 (i.e., 38 per group) to yield at least 86.5% power, assuming at least 100 subjects would return on day 3 in our study.

### Intervention Arms

**Interventions.** There were two intervention arms: aware [AW] and unaware [UW] groups in this study. In the AW (Vivofit aware) group, participants were able to see their step counts in real-time on the device screen. In the UW (Vivofit unaware) group, the device screen was completely covered with black ink so that participants would not be able to see the number of steps. Participants in both intervention arms were asked to wear a wearable technology (Garmin Vivofit) like a bracelet for PA tracking (step count, hours of sleep) on the non-dominant arm continuously 24 hours for 2 days. This wearable technology used was a commercially available PA tracking device that has the feature of revealing the number of steps taken. The device was waterproof and had a 2-year battery life; therefore, participants were asked to keep the device on at all times. The steps and hours of sleep data were extracted from Garmin Connect, an internet platform that linked with each device. The differences between the two arms were the ability to see the step count numbers screen.

**Control.** In the control/usual care (C) group, participants received their usual care and completed the study questionnaire package.

### Measures

**Feasibility.** The feasibility of using the wearable device was measured by the number of reported adverse events (e.g, allergic reaction to the band, discomfort, and device malfunction), participants’ acceptability and satisfaction, and participants’ compliance in wearing the device. Participants in the AW and UW groups were asked to complete the open-ended questions to measure their experience, acceptability, and satisfaction with the device.

#### Demographics and Outcomes

*Demographic and clinical information.* Participants were asked to complete the demographic form developed by the researchers to collect information about age, gender, education, marital status, religion, family history of diabetes, and income. The medical history was collected from the medical record review. Weight (kg), height (m), waist circumference (cm), and blood pressure were also collected.

*Self-management behaviors* were measured by the revised summary of diabetes self-care activity (SDSCA)-Thai version. This instrument is a 17-item, 7-point Likert self-report, which assesses the diabetes self-care behaviors of individuals, to include eating activities (5 items), physical activity (2 items), medication adherence (3 items), blood glucose testing (2 items), and foot care (5 items). The total score ranged from 0 to 119. The higher the score, the better the self-care behavior of the individual. The Thai version of this instrument was tested in 124 Thai with T2DM and found to have acceptable reliability (internal consistency *R* = 0.23–0.80) (Wattanakul, 2012).

*Symptom experiences.* Fatigue and pain severity were measured by a visual numeric scale for each symptom. Participants were asked to rate their symptom experience in the past 2 weeks on a scale of 0 (no symptom) to 10 (severe symptom). A higher score indicated more severe symptoms.

*Self-efficacy* was measured by the Diabetes Self-Efficacy Scale (DSES), which was developed by the Stanford Self-Management research Program (Self-management Resource Center, 2020). This 8-item self-reported instrument was a 10-point Likert scale questionnaire, which measured the person’s confidence level in diabetes self-management. A higher score means a better confidence level in self-management. The DSES was tested and found to have an acceptable internal consistency (Cronbach’s alpha > 0.8) (Lorig, Ritter, Villa, & Armas, 2009; Ritter & Lorig, 2014; Ritter, Lorig, & Laurent, 2016).

*Quality of life* was measured by the General Health Short Form 12 (SF-12), which was modified from the original SF-36. These 12 items were used to measure eight physical and mental health domains: General Health (GH), Physical Functioning (PF), Role Physical (RP), Body Pain (BP), Vitality (VT), Social Functioning (SF), Role Emotional (RE), and Mental Health (MH). These domains were summarized into a physical health component (average of GH, PF, RP, and BP) and a mental health component (average of VT, SF, RE, and MH). A higher score means a better quality of life. This instrument has been tested with many populations including people in Thailand, where it has proved its conciseness, comprehensiveness, reliability (Cronbach’s alpha coefficients of 0.72–0.89), test-retest reliability (*r = *0.73–0.86), validity, and cross-cultural applicability (Chariyalertsak et al., 2011).

### Data Collection Procedures

Once the consent form was signed, participants were asked to complete a questionnaire package consisting of (1) demographic form, (2) self-management behaviors, (3) self-efficacy, (4) symptoms experiences, and (5) quality of life. Participants were randomly assigned to one of the three groups. On day 3 the participants in intervention groups were asked to return the device to the clinic. In this study, we did not ask the participants to use the phone application or the internet platform that links with the device, which makes it easy for patients to use with minimal training required. The Vivofit was synchronized with the research team’s laptop computer and participants were shown and provided instructions on how to use these devices (the device is worn like a bracelet) (Figure 1).

### Ethical Consideration

Potential participants were approached during their clinic visits and received a verbal and written explanation of the study objectives and methods. They were encouraged to ask researchers’ questions about the study. Recruitment was followed by study enrollment. Data was collected after the consent form was signed. The study was approved by the University of Nevada Las Vegas IRB and the Faculty of Nursing, Chiang Mai University Research Ethics Committee (STUDY CODE: EXP-133-2557).

### Randomization

Participants were randomly assigned to one of the three groups by selecting one of the three sealed envelopes that contact the group number. Participants who select enveloped with group 1 were in the AW (Vivofit aware) group, group 2 was in the UW (Vivofit unaware) group, and group 3 were in the C (usual care) group.

### Data Analysis

To examine the association between PA level measured by the wearable device and demographic, anthropomorphic, clinical, and psychosocial (self-efficacy, self-management, and symptoms) measures, bivariate correlations (Pearson’s *r*) were conducted. In addition, the significance of the difference between the two correlation coefficients by group (i.e., AW [Vivofit aware] group vs. UW [Vivofit unaware] group) was tested using the Fisher *r*-to-*z* transformation, to compare the correlation between each of the above measures and PA level measured by the two wearable devices.

General linear models were conducted to evaluate the difference in clinical and psychosocial measures among the three groups after adjusting for the effects of age and gender. Tukey’s method was used for pairwise comparisons in the general linear models to control for the familywise error rate (FWER) at 5%. All statistical analyses were conducted using the statistical analysis software SAS version 9.4 (SAS Institute Inc., Cary, NC); the significance level for all tests was set at 5%.

## Results

One hundred and fourteen Thai participants diagnosed with T2DM, ages 39–70 years old (mean ± SD = 56.5 ± 7.2) were included in this analysis. The majority were female (*n* = 86, 69%) and married (*n* = 103, 82%). Approximately 90% had primary school education. More than 68% were overweight and obese (BMI < 22.9). Their fasting blood glucose (FBS) ranged from 70 to 329 (mean ± SD = 143.2 ± 45.3). The hemoglobin A1C ranged from 5.3% to 11.5% with a mean of 8.3% (SD = 1.8) (Table 1).

**Table 1:** Demographic and Clinical Information

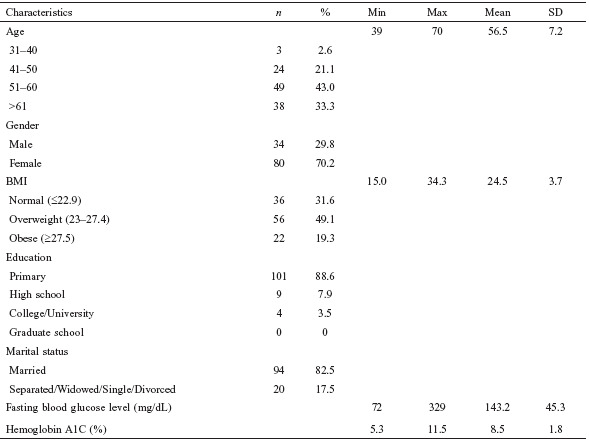

### Feasibility of the Wearable Device

Only participants in the AW and UW groups (*n* = 76) were asked about their experience of using the wearable device.

**Adverse Events.** No adverse events (discomfort, allergic reactions, or device malfunctions) were reported for both groups (Table 2).

**Table 2:** Feasibility of the Wearable Device (n = 76)

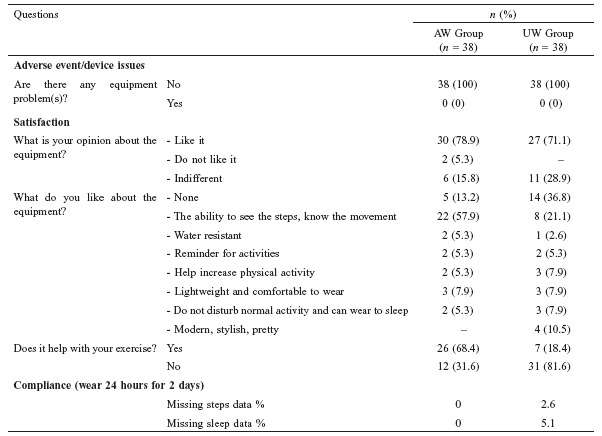

**Acceptability and Satisfaction.** The majority of participants in both groups reported that they liked using the device (79% of the AW group and 71% of the UW group). More than 55% of participants in the AW group liked the device because of the ability to see the step count; the majority of the UW group (37%) did not answer this question. Nearly 70% of participants in the aware group felt that this device helped increase their exercise; 82% of participants in the unaware group did not think that the device increased their exercise (Table 2).

**Compliance.** The compliance for wearing the device 24 hours for 2 full days was measured by the percentage of a missing step and sleep data. The compliance rate was high for both groups. Only participants in the UW removed the device (missing data range from 5.1% to 2.6%) (Table 2).

### Differences in Physical Activity and Sleep between the Aware and Unaware Groups

No significant difference was found for the number of steps and hours of sleep between the AW and UW groups; however, the AW group had slightly higher mean steps than the UW group on both days 1 and 2 (Table 3). More than 63% of participants who received a Garmin Vivofit in both the AW and UW groups had an average step count greater than 10,000 steps/day. More participants in the AW group (41%) increased their step on day 2 than the UW group (37%).

**Table 3:** Comparison Results for Wearable Device Step Counts and Hours of Sleep Data between the Vivofit Aware and Vivofit Unaware Groups (n = 76)

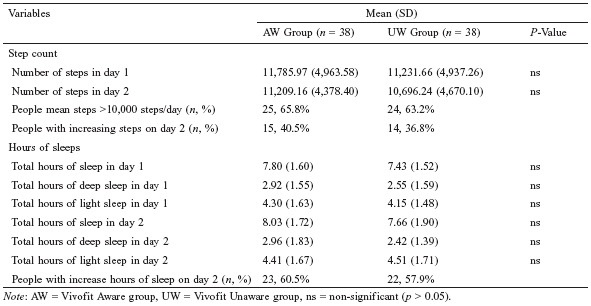

Participants in the AW group had longer sleep hours than the participants in the UW group on both days; however, these differences were not statistically significant. More participants in the AW groups (61%) had increasing hours of sleep on day 2 than the UW group (58%).

### Differences in Symptoms, Self-Management, and Quality of Life among the Three Groups

Pairwise comparisons using Tukey’s method among the three groups did not show significant differences in self-management behaviors, symptoms, or self-efficacy (Table 4). The participants who wore the device (both the unaware and aware groups) had slightly higher self-management behaviors and self-efficacy and lower severity of symptoms than those in the control group. Only the general health domain of quality of life was significantly different among the three groups (*p* < 0.008).

**Table 4:** Comparison Results across Three Groups for Self-management Behaviors, Symptom Experience, Self-efficacy, and Quality of Life (n = 114)

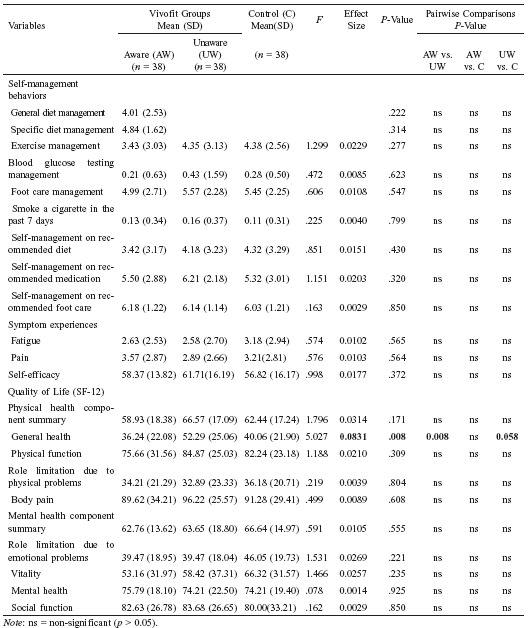

## Discussion

Self-monitoring is a foundation of many behavioral change interventions, including smoking, diet, and PA (Rossi et al., 2018; Sanders et al., 2016; Schrager et al., 2017; Sharman, Zhussupov, Sharman, & Kim, 2020). For PA, the pedometers and electronic wearable devices (e.g., Fitbit, Jawbone, Garmin Vivofit) became popular methods of self-monitoring. A systematic review and meta-analysis found that among 12 clinical trials from the United States, Canada, Europe, and Australasia (1,458 participants), the use of a pedometer substantially increased free-living PA among patients with T2DM (Baskerville, Ricci-Cabello, Roberts, & Farmer, 2017). One of the factors that influenced the PA changes was the capacity for self-monitoring of the device (Tudor-Locke & Lutes, 2009). Pedometers and accelerators had been used in Thailand in many PA programs; however, many of the programs were specifically designed for children and young adults (Konharn, Santos, & Ribeiro, 2015; Morinaka, Limtrakul, Makonkawkeyoon, & Sone, 2012; Oftedal et al., 2017). Our study provides additional evidence for the feasibility of the pedometer commercial wearable device for self-monitoring and as reminding tools for middle age and older adults with chronic conditions. The 76 participants who wore the device for 2 days did not report any allergic reaction or discomforts, excellent compliance (less than 10% missing data), and expressed high satisfaction rates (75%).

Our results showed that the ability to see the step count was one of the main reasons why participants liked the device. We noted that the majority of the participants (90%) had primary school education levels, which may have impacted their ability to obtain, read, understand, and communicate about health-related information or health literacy. A study reported that health literacy level was associated with the self-reported PA but not the objective steps count/day (Al Sayah, Johnson, & Vallance, 2016). For our study, the focus was on the feasibility and acceptability of the device among this population by providing simple instruction on how to wear the device and remove the device (if needed), and the meaning of the numbers on the screen (AW group only). Our participants were able to comply with this instruction, which suggested that the amount of instruction was appropriate and different health literacy friendly.

Among 76 participants who worn the Garmin Vivofit, no statistical differences were found on daily steps and sleep hours measured by the wearable device between the AW and UW groups. However, nearly 70% of participants who could see the daily steps felt that they had increased their exercise; 82% of the participants who could not see the steps felt that the device did not help with their exercise. Comparing the step counts between days 1 and 2, we found that more participants who could see their steps (AW group) increased their steps and had a longer sleep duration on day 2 than the UW group. Consistent with another study (Wang et al., 2016), our results indicated that the instant display of the PA performance had an effect on participants’ awareness of PA levels and may have influenced their self-regulation to activate changes in steps/day and behaviors.

Although no significant differences were found among the three groups on self-management behaviors, symptoms, and self-efficacy, participants who received the wearable device in both the AW and UW groups had slightly higher self-management behaviors, self-efficacy, and lower symptoms. We found that participants in the control and unaware groups had a higher quality of life in both physical and mental health components. The pairwise comparison showed that the general health domain of quality of life in the UW groups was significantly higher than the AW and control groups. This unexpected result could have been affected by the duration (2 days) of the wearable device usage and the one-time collection of the questionnaires, which may not have captured the impact of the wearable device on quality of life.

## Limitations and Recommendations

Although these findings suggest the potential benefits of the instant display of the step counts on the wearable device among individuals with diabetes in Thailand, this study has some limitations. First, the duration of the device usage was only 2 days. The use of the device for only 2 days allowed us to investigate the possibilities of usage issues, and participants’ acceptability and satisfaction with the device. The optimal program duration, PA goal setting, and follow-up patterns should be further investigated to determine the impact of the program on the change in PA and health behaviors. Second, we were unable to measure the baseline steps and sleep hours without the device for the within-subject comparison; however, we found that wearing the device and seeing the daily step count may have had some impacts on participants’ awareness of their PA level and may have activated their behavior changes. Future intervention studies should include the impact of the device on health behaviors into consideration.

Another limitation is that our study did not include different health literacy levels. Our intervention did not include instructions for the data interpretation and the PA goal setting. The devices’ data were extracted from the web-based platform by the research team. While this process is low health literacy friendly, we were unable to measure the magnitude of the intervention impact on the PA. Detailed instructions, clear PA goals, and the interpretation of the data to facilitate self-monitoring and self-management should be included in future intervention studies. The individuals’ health literacy and its’ impact on the effectiveness of the program should be examined.

## Summary

In conclusion, our study provides supportive evidence of commercially available wearable devices to measure PA and sleep hours among patients with chronic conditions in the community in Thailand. With its long battery life, the device was easy to install and manage. The screen that showed step counts was found to be a useful feature of this wearable device; however, we were unable to assess the change of the PA and sleep due to the limited usage duration. Future studies should be done to examine the effect of these immediate feedback features on behavioral changes over a longer duration. Although the device was lightweight, had long battery life, and was user friendly, the reliability of this device should be tested by comparison with the gold standard PA and sleep measurement.

## Acknowledgments

We thank our participants for their contribution to this study. We also thank nursing students from the Faculty of Nursing, Chaing Mai University, Thailand, and doctors, nurses, and community health volunteers from Nakornping and Hangdong Hospitals, Chiang Mai, Thailand who facilitated the recruitment and collected the data. We appreciate Mr. Martin F. Blair from Johns Hopkins School of Nursing editing service for the English language editing.

## Declaration of Conflicting Interests

All authors certify that we have no affiliations with or involvement in any organization or entity with any financial interest or non-financial interest in the subject matter or materials discussed in this study.

## Funding

The study was funded by the Tony & Renee Marlon Angel fund, School of Nursing, University of Nevada Las Vegas-Dean Research Support, and the Faculty Research Support fund from Faculty of Nursing, Chiang Mai University, Thailand.

Dr. Li’s time is supported in part by the University of Rochester, CTSA award number UL1 TR002001 from the National Center for Advancing Translational Sciences of the National Institutes of Health.
